# 7-T MRI intratumoral susceptibility signals reflect biomarker status in gliomas

**DOI:** 10.1186/s41747-026-00696-0

**Published:** 2026-04-02

**Authors:** Fengwei Yu, Ke Li, Zilong Li, Suyi Zhou, Wei Chen, Chaodong Xiang, Jiafei Chen, Zhentao Zuo, Zhiming Zhen, Wei Chen

**Affiliations:** 1 7T Magnetic Resonance Translational Medicine Research Center, Department of Radiology, Southwest Hospital, Third Military Medical University (Army Medical University), Chongqing, China; 2 Department of Radiology, Southwest Hospital, Third Military Medical University (Army Medical University), Chongqing, China; 3 MR Research Collaboration Team, Siemens Healthineers Ltd., Guangzhou, China; 4 School of Medicine, Chongqing University, Chongqing, China; 5 State Key Laboratory of Brain and Cognitive Science, Institute of Biophysics, Chinese Academy of Sciences, Beijing, China; 6 University of Chinese Academy of Sciences, Chinese Academy of Sciences, Beijing, China

**Keywords:** Biomarkers, Glioma, Multiparametric magnetic resonance imaging, Pathology (molecular), Precision medicine

## Abstract

**Objective:**

To evaluate whether 7-T susceptibility-weighted imaging (SWI) can predict glioma’s histological grade, Ki-67 labeling index (LI), isocitrate dehydrogenase 1 (IDH1) mutation, 1p/19q co-deletion, telomerase reverse transcriptase (TERT) promoter mutation, and O-6-methylguanine-DNA-methyltransferase (MGMT) promoter methylation status of gliomas.

**Materials and methods:**

We retrospectively analyzed 7-T SWI in 60 patients with glioma. The Mann–Whitney *U* test compared the intratumoral susceptibility signals (ITSS) grade across molecular markers, with ITSS defined as fine linear or dot-like low signal areas on SWI. Predictive efficacy was assessed using receiver operating characteristic (ROC) analysis and multivariate logistic regression models. Path analysis evaluated the relationships between ITSS grade and molecular markers.

**Results:**

Gliomas with high ITSS grade showed higher histological grade, Ki-67 LI, and TERT mutation rates compared to those with low ITSS grade, mostly being wild-type gliomas. ITSS grade predicted the histological grade, Ki-67 LI, and TERT status (area under the ROC curve = 0.769‒0.817). Multivariate logistic regression analysis identified Ki-67 LI and TERT status as independent predictors of high ITSS grade. Path analysis indicated direct effects of Ki-67 LI and TERT mutation on ITSS grade, and an indirect effect of IDH1 mutation on ITSS grade mediated through Ki-67 LI.

**Conclusion:**

7-T SWI-derived ITSS grade predicts histologic grade, Ki-67 LI, and TERT promoter mutation status in gliomas. Ki-67 LI and TERT mutation exert relatively independent effects on ITSS grade and allow reverse inference of their status from SWI, whereas IDH1 mutation influences ITSS grade indirectly via Ki-67 LI.

**Relevance statement:**

This study establishes a connection between preoperative imaging and molecular glioma pathology via 7-T SWI. It helps to reveal the *in vivo* characteristics of pathology and promotes collaboration among radiologists, pathologists, and clinicians, which a great clinical potential.

**Key Points:**

- A 7-T susceptibility-weighted MRI–based intratumoral susceptibility signal (ITSS) grading system enables precise detection of glioma microbleeds and neovascularization.
- 7-T susceptibility-weighted MRI–derived ITSS grade noninvasively predicts histologic grade, Ki-67 labeling index, and telomerase reverse transcriptase (TERT) promoter mutation status in gliomas.
- Path analysis suggested that molecular markers relate to ITSS grade through distinct pathways, with Ki-67 and TERT exerting direct effects and isocitrate dehydrogenase 1 influencing ITSS grade indirectly.

**Graphical Abstract:**

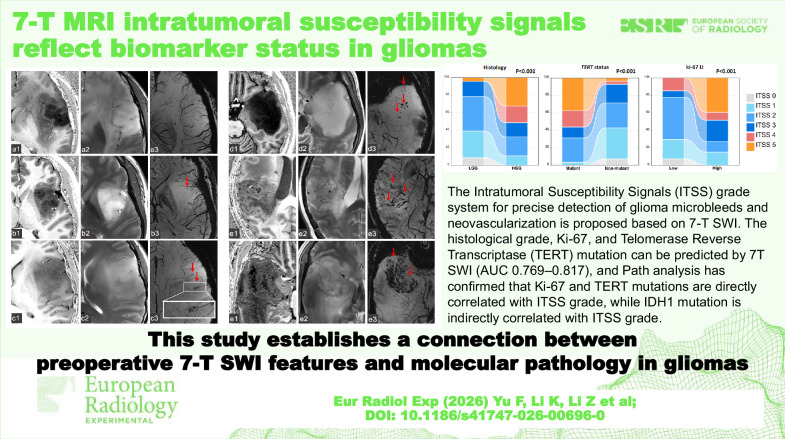

## Background

The 2021 World Health Organization (WHO) classification of the central nervous system tumors [1] integrates histopathological and molecular characteristics to refine glioma diagnosis, grading, personalized treatment strategies, and prognostic evaluation. Key molecular markers strongly influence patient outcome. High Ki-67 labeling index (LI) indicates aggressive tumor cell proliferation and portends poor prognosis [2]. Telomerase reverse transcriptase (TERT) mutation status reflects increased invasiveness and poor prognosis [3]. In contrast, isocitrate dehydrogenase 1 (IDH1) mutation, 1p/19q chromosomal deletions or oxygen-6-methylguanine-DNA-methyltransferase (MGMT) associate with more favorable prognosis and longer survival [4–7]. However, the determination of these molecular features often requires surgical resection or biopsy, which limits repeated evaluation and carries procedural risk. Neuro-oncology therefore seeks noninvasive imaging biomarkers that dynamically track glioma biology.

Magnetic resonance imaging (MRI) serves as a noninvasive imaging technique and plays an important role in the clinical diagnosis and treatment of gliomas. Multiparametric 3-T MRI—combining structural, functional, and metabolic sequences—already supports preoperative grading and molecular marker prediction in gliomas [8–10]. Among these techniques, susceptibility-weighted imaging (SWI) has attracted widespread attention due to its unique sensitivity to paramagnetic substances (such as hemosiderin and deoxygenated blood). It can sensitively detect differences in tissue susceptibility within gliomas and detect neovascularization (venous blood), microbleeds, and mineralization within gliomas [11, 12]. At 3 T, intratumoral susceptibility signals (ITSS), defined as fine linear or dot-like hypointense foci on SWI, provide a semiquantitative grading of microbleeds and abnormal vasculature [13]. Existing studies have shown that multimodal imaging, including 3-T SWI, has demonstrated significant clinical application potential in predicting the histological grade, Ki-67 labeling index (LI), IDH1 mutation, and MGMT promoter methylation of gliomas [8, 9, 14]. Nevertheless, these approaches often require complex multiparametric protocols and advanced postprocessing, which may limit routine adoption.

Ultra-high field 7-T SWI enhances signal-to-noise ratio, spatial resolution, and susceptibility contrast for T2*-weighted sequences [15]. These advantages sharpen the visualization of microvasculature and microhemorrhages and may improve the detection of ITSS. Recent studies employing 7-T quantitative blood oxygen level-dependent MRI further demonstrate the potential of ultra-high-field MRI to assess hypoxia dynamics and treatment response in glioma [16], collectively highlighting its broader utility in characterizing the glioma microenvironment. A simple, visually based ITSS grading system on 7-T SWI could therefore exploit these technical gains while remaining easy for clinicians to apply. Such an approach may capture tumor microvascular architecture and microbleeding patterns that reflect underlying molecular alterations, and thus provide an intuitive, *in vivo* surrogate of glioma pathology.

Therefore, we propose to adopt a method that is easy for clinicians to observe and describe in a short time, namely the ITSS grade method based on high-quality 7-T SWI imaging data, to evaluate the imaging features of microbleeds and neovascularization in gliomas. It is important to emphasize that the primary goal of such imaging biomarkers is to provide a noninvasive supplement for diagnostic workup and preoperative planning. This study aims to further predict the status of key molecular markers, such as Ki-67 LI, TERT promoter mutations, and explore the correlation between preoperative imaging features and molecular pathology.

## Materials and methods

### Patients

The study protocol was approved by the Ethics Committee of the First Affiliated Hospital of the Army Medical University (KY2024109), all procedures complied with institutional guidelines and regulations, and all the participants provided written informed consent.

We retrospectively analyzed the results of 60 adult patients (27 males, 33 females; age mean ± standard deviation 48.42 ± 11.61 years; age range 24–72 years) diagnosed with gliomas and treated surgically at our Hospital from March 2023 to May 2025. The inclusion criteria were: (1) gliomas confirmed pathologically according to the 2021 WHO classification of tumors of the central nervous system [1]; and (2) age 18–80 years. The exclusion criteria were: (1) patients with previous diagnosis of any brain tumor, no history of brain biopsy or surgery, or history of brain radiotherapy or chemotherapy; (2) patients with severe infections or strokes; (3) patients who did not undergo complete pathological molecular marker testing after surgery; and (4) patients with severe motion artifacts or foreign body artifacts.

### MRI protocol and ITSS grade

All MRI examinations were performed on a 7-T MAGNETOM Terra scanner (Siemens Healthcare). The detailed protocol is presented in the Supplementary Materials.

Two radiologists, each with 5 years of experience in neuroimaging, independently graded ITSS on 7-T SWI. A senior neuroradiologist with 25 years of experience re-evaluated discrepant cases and provided the final consensus grade.

**Fig. 1:**
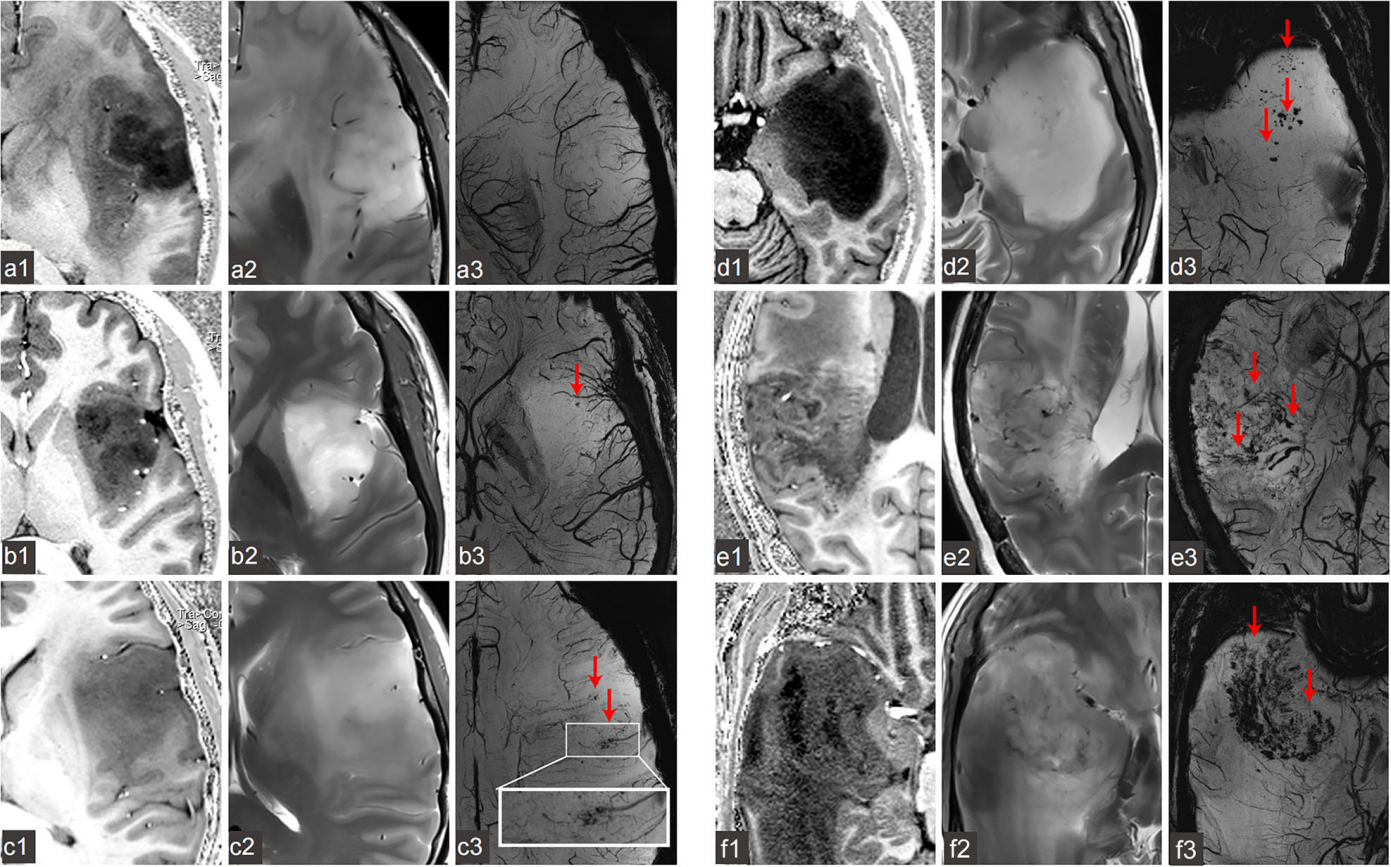
Intratumoral susceptibility signals (ITSS) in different grades of glioma (indicated by arrows). Demonstrated through three sequences: T1-weighted imaging (**a1**, **b1**, **c1**, **d1**, **e1**, **f1**), T2-weighted imaging (**a2**, **b2**, **c2**, **d2**, **e2**, **f2**), and SWI (**a3**, **b3**, **c3**, **d3**, **e3**, **f3**). **a1**–**a3** Astrocytoma WHO II, ITSS 0 (ITSS = 0); **b1**–**b3** Oligodendroglioma WHO II, ITSS 1 (1 ≤ ITSS ≤ 10); **c1**–**c3** Astrocytoma WHO III, ITSS 2 (11 ≤ ITSS ≤ 35); **d1**–**d3** Glioblastoma WHO IV, ITSS 3 (36 ≤ ITSS ≤ 50); **e1**–**e3** Glioblastoma WHO IV, ITSS 4 (51 ≤ ITSS ≤ 85); **f1**–**f3** Glioblastoma WHO IV, ITSS 5 (ITSS > 85)

**Fig. 2:**
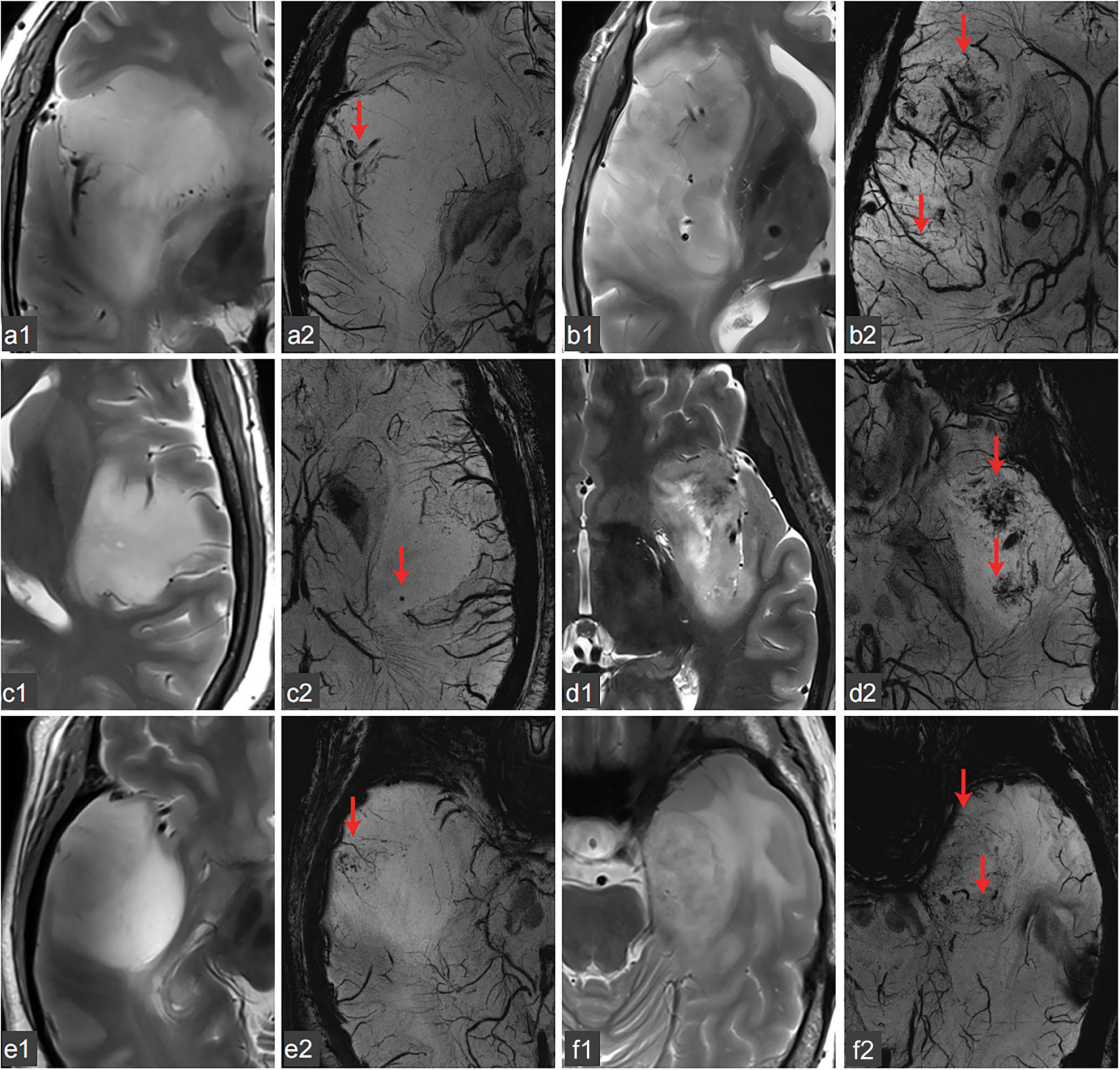
ITSS presentation is associated with the expression status of different molecular markers (as indicated by arrows). Demonstrated through two sequences: T2-weighted imaging (**a1**, **b1**, **c1**, **d1**, **e1**, **f1**) and SWI (**a2**, **b2**, **c2**, **d2**, **e2**, **f2**). **a1**, **a2** Astrocytoma WHO III, low Ki-67 expression, ITSS 2; **b1**, **b2** Oligodendroglioma WHO III, high Ki-67 expression, ITSS 4; **c1**, **c2** Astrocytoma WHO II, no TERT promoter mutation, ITSS 2; **d1**, **d2** Oligodendroglioma WHO II, TERT promoter mutation, ITSS 4; **e1**, **e2** Astrocytoma WHO III, IDH1 mutation, no TERT promoter mutation, ITSS 2; **f1**, **f2** Glioblastoma WHO IV, no IDH1 mutation, no TERT promoter mutation, ITSS 3

ITSS was defined as fine linear or dot-like low signal areas on SWI sequences, which could be categorized into three types based on morphological features: punctate structure clusters, linear structure clusters, and mixed structures [17]. To ensure consistency with prior studies and to maintain clinical practicality, referring to the 3-T SWI ITSS grade scheme [17], we used minimum intensity projection technology to semiquantitatively evaluate 7-T SWI, quantified ITSS on the slice with the largest cross-sectional area of the glioma parenchyma, and redefined the grading criteria based on the quantification results of ITSS. Instructions for the formulation of grading criteria can be found in the Supplementary Materials. The new grading scheme has been defined as follows: grade 0 = no ITSS; grade 1 = 1–10 dot-like or linear ITSS; grade 2 = 11–35 dot-like or linear ITSS; grade 3 = 35–50 dot-like or linear ITSS; grade 4 = 50–85 dot-like or linear ITSS; grade 5 = more than 85 dot-like or linear ITSS (Figs. 1 and 2).

### Discrimination of calcifications

Because SWI is sensitive to both diamagnetic substances, such as calcifications, and paramagnetic substances, such as deoxyhemoglobin and hemosiderin [18], we ensured that ITSS grading reflected tumoral vasculature and microhemorrhages rather than calcifications. During grading, the readers always reviewed SWI together with the corresponding original phase images. As illustrated in Supplementary Fig. S3, diamagnetic calcifications typically appear hyperintense on phase images, whereas paramagnetic blood products appear hypointense. Readers used these phase characteristics, together with morphology, to identify and exclude calcific foci from the ITSS count, so that the graded signals primarily reflected vascular phenomena of interest. The high spatial resolution of 7-T SWI further facilitated this discrimination.

### Molecular pathology detection

All molecular data were obtained from clinical pathology reports. The panel included Ki-67 LI, IDH1 mutation status, 1p/19q co-deletion status, TERT promoter mutation status, and MGMT promoter methylation status. Pathology laboratories processed and interpreted all tissue specimens according to the technical specifications and reporting standards of the National Comprehensive Cancer Network (NCCN) and the Consortium to Inform Molecular and Practical Approaches to CNS Tumor Taxonomy (cIMPACT-NOW) [19, 20]. The detailed information on the experimental methods has been described in the Supplementary Materials section.

### Statistical analysis

We used the Mann–Whitney *U* test to compare ITSS grades between groups defined by histologic grade and by each molecular marker. We then evaluated the predictive performance of the ITSS grade for individual molecular markers using receiver operating characteristic (ROC) analysis and logistic regression models. For each prediction task, we calculated sensitivity, specificity, and area under the ROC curve (AUROC).

Univariable logistic regression first tested the association between ITSS grade and each marker. We then performed multivariable logistic regression with a forward stepwise selection procedure to identify independent predictors. Variables entered the model when the *p*-value was less than 0.05 and were removed when the *p*-value exceeded 0.10. We reported odds ratios (ORs) with corresponding 95% confidence intervals (CIs) and presented these results in forest plots.

To explore the network of relationships among ITSS grade and multiple molecular markers, we conducted path analysis, which allowed us to model direct and indirect effects and to infer potential causal pathways. We performed Mann–Whitney *U* tests and path analysis using SPSS (IBM Corp., Armonk, NY, USA), generated ROC curves using R software (version 4.3.0), and carried out multivariable logistic regression and forest plot visualization using Zstats version 1.0 (www.zstats.net). A two-sided *p*-value less than 0.05 indicated statistical significance.

Interobserver agreement between the two primary radiologists was quantified using the intraclass correlation coefficient for absolute agreement, based on a two-way random-effects model, with values interpreted as follows: < 0.50, poor; 0.50–0.75, moderate; 0.75–0.90, good; and > 0.90, excellent agreement.

## Results

### Association between ITSS grade and histological grade, Ki-67 LI, IDH1, MGMT, TERT, and 1p/19q status

Clinical, molecular pathological, radiological, and SWI data were collected from 60 patients with glioma. The patient characteristics under the six-tier ITSS classification scheme are presented in Table 1. Additionally, patient characteristics under the four-tier ITSS grade scheme are presented in Supplementary Table S3. The interobserver agreement for ITSS grading between the two neuroradiologists was excellent, with an intraclass correlation coefficient of 0.98 (95% confidence interval: 0.97–0.99). In cases with discrepant grades, the senior neuroradiologist adjudicated and provided the final ITSS grade.

**Table 1:** Clinical and SWI characteristics of glioma patients with different molecular status

Characteristics	Histology			IDH1 status			Ki-67 LI		
	LGG	HGG	*p*-value	+	-	*p*-value	Low	High	*p*-value
Age	46.41 ± 10.49	49.97 ± 12.12	0.235	47.14 ± 10.10	51.00 ± 13.47	0.134	48.89 ± 11.05	48.44 ± 12.18	0.727
Sex (males/females)	13/10	14/23	0.157	18/19	9/14	0.471	14/13	13/20	0.335
ITSS grade			**< 0.001**			**0.032**			**< 0.001**
ITSS 0	2	0		2	0		2	0	
ITSS 1	7	4		4	7		6	5	
ITSS 2	9	8		14	3		13	4	
ITSS 3	4	6		5	5		2	8	
ITSS 4	0	7		4	3		4	3	
ITSS 5	1	12		5	8		0	13	

Characteristics	MGMT status			1p/19q status			TERT status		
	M	U	*p*-value	Codeletion	Non-codeletion	*p*-value	+	-	*p*-value
Age	49.49 ± 10.95	47.00 ± 12.83	0.599	50.35 ± 8.97	47.95 ± 12.51	0.445	51.00 ± 11.11	45.85 ± 11.69	0.072
Sex (M/F)	19/21	8/12	0.582	8/9	19/24	0.840	16/16	11/17	0.405
ITSS grade			0.422			0.084			**< 0.001**
ITSS 0	2	0		0	2		0	2	
ITSS 1	6	5		1	10		1	10	
ITSS 2	14	3		6	11		9	8	
ITSS 3	6	4		1	9		4	6	
ITSS 4	5	2		4	3		6	1	
ITSS 5	7	6		5	8		12	1	

Bold values indicate statistical significance *p* < 0.05

Binary data are shown as counts; age is expressed as mean ± standard deviation

IDH1 status “+”: IDH1 mutation; IDH1 status “-”: no IDH1 mutation

Low expression: Ki-67 LI < 10%; high expression: Ki-67 LI ≥ 10%

TERT status “+”: TERT promoter mutation; TERT status “-”: no TERT promoter mutation

*HGG* High-grade glioma, *ITSS* Intratumoral susceptibility signals, *IDH* Isocitrate dehydrogenase, *LGG* Low-grade glioma, *LI* Labeling index, *MGMT* O-6-Methylguanine-DNA methyltransferase, *M* MGMT promoter methylation, *U* MGMT promoter unmethylation, *TERT* Telomerase reverse transcriptase

**Fig. 3:**
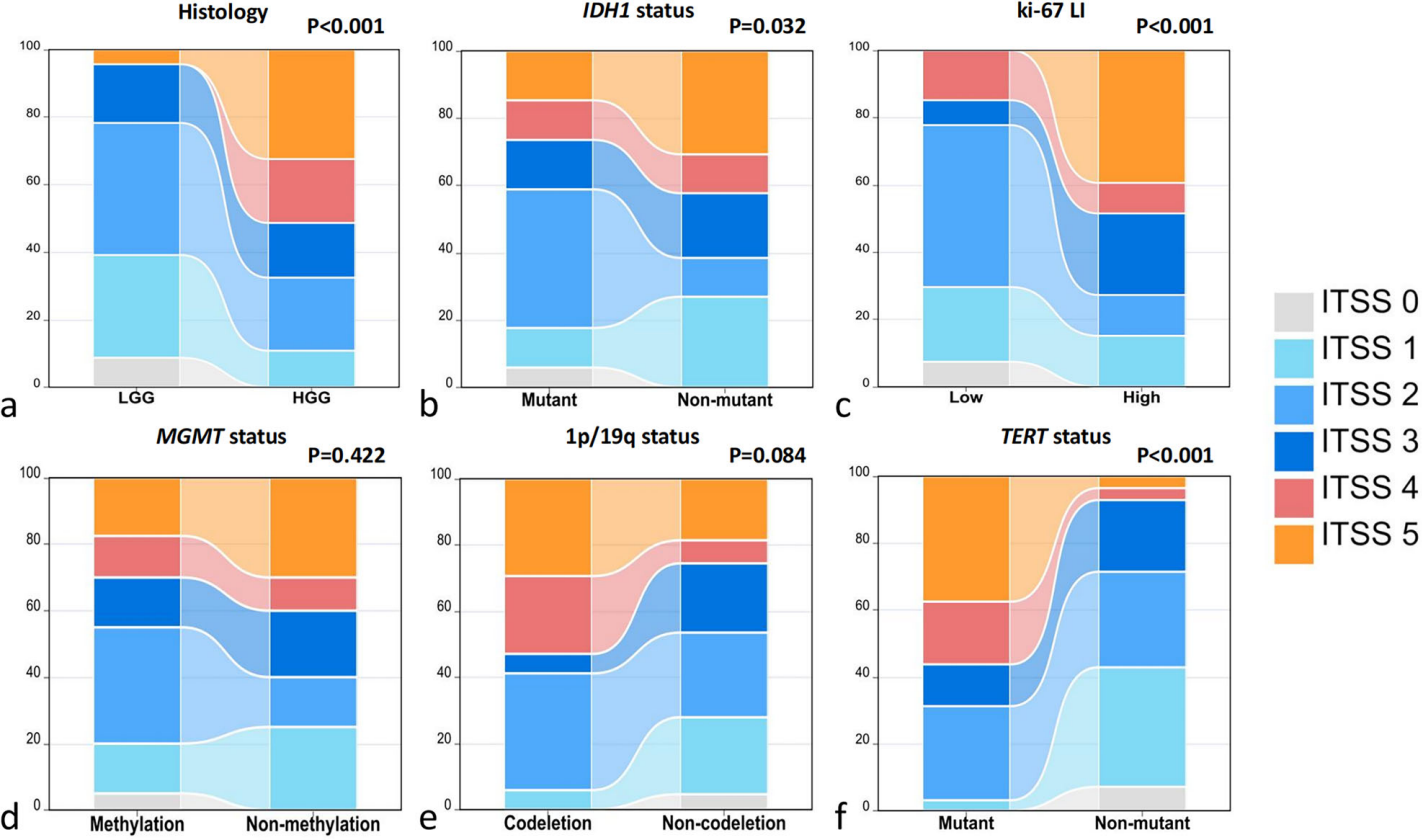
Histograms (analyzed using the Mann–Whitney test) show the correlation between ITSS grade with histological grade, Ki-67 LI, status of 1p/19q, IDH1, MGMT, and TERT. **a** The ITSS grade of high-grade gliomas is significantly higher than that of low-grade gliomas (*p* < 0.001). **b** There is a significant difference between ITSS grade and IDH1 expression status (*p* = 0.032). **c** The ITSS grade of gliomas with high Ki-67 expression is significantly higher than that of gliomas with low Ki-67 expression (*p* < 0.001). **d** There is no significant difference between ITSS grade and MGMT expression status (*p* = 0.422). **e** There is no significant difference between ITSS grade and 1p19q expression status (*p* = 0.084). **f** The ITSS grade of gliomas with TERT promoter mutation is significantly higher than that of gliomas without TERT promoter mutation (*p* < 0.001)

Our study showed that the ITSS grade of high-grade glioma (HGG) was significantly higher than that of low-grade glioma (LGG) (*p* < 0.001). Similarly, the ITSS grade of gliomas with IDH1 mutation was much lower than that of IDH1 wild-type gliomas (*p* = 0.032), the ITSS grade of gliomas with high Ki-67 expression was much higher than that of gliomas with low Ki-67 expression (*p* < 0.001), and the ITSS grade of gliomas with TERT mutation was also much higher than that of gliomas without TERT mutation (*p* < 0.001). However, there was no significant difference between ITSS grade and MGMT methylation status or 1p/19q chromosomal codeletion status (*p* = 0.422 and *p* = 0.084, respectively) (Fig. 3).

In addition, we separately analyzed the association between ITSS grade and Ki-67 LI, IDH1, MGMT, TERT, and 1p/19q status in both the LGG and HGG groups (Table 2). In the HGG group, gliomas with 1p/19q chromosomal codeletion demonstrated significantly higher ITSS grade than those without 1p/19q chromosomal codeletion (*p* = 0.019).

**Table 2:** ITSS characteristics of IDH1, Ki-67 LI, MGMT, 1p/19q, and TERT status in different grades of gliomas

Characteristics	IDH1 status			Ki-67 LI			MGMT status			1p/19q status			TERT status		
	+	–	*p*-value	Low	High	*p*-value	M	U	*p*-value	Codeletion	Non-codeletion	*p*-value	+	–	*p*-value
**LGG**															
ITSS grade			0.647			**0.017**			0.737			0.092			0.092
ITSS 0	2	0		2	0		2	0		0	2		0	2	
ITSS 1	6	1		6	1		5	2		1	6		1	6	
ITSS 2	8	1		9	0		9	0		6	3		6	3	
ITSS 3	4	0		1	3		3	1		1	3		1	3	
ITSS 4	0	0		0	0		0	0		0	0		0	0	
ITSS 5	1	0		0	1		1	0		1	0		1	0	
**HGG**															
ITSS grade			0.508			0.222			0.742			**0.019**			< **0.001**
ITSS 0	0	0		0	0		0	0		0	0		0	0	
ITSS 1	1	3		0	4		1	3		0	4		0	4	
ITSS 2	6	2		4	4		5	3		0	8		3	5	
ITSS 3	1	5		1	5		3	3		0	6		3	3	
ITSS 4	4	3		4	3		5	2		4	3		6	1	
ITSS 5	4	8		0	12		6	6		4	8		11	1	

Bold values indicate statistical significance *p* < 0.05

IDH1 status “+”: IDH1 mutation; IDH1 status “-”: no IDH1 mutation

Low expression: Ki-67 LI < 10%; high expression: Ki-67 LI ≥ 10%

TERT status “+”: TERT promoter mutation; TERT status “-”: no TERT promoter mutation

*HGG* High-grade glioma, *ITSS* Intratumoral susceptibility signals, *IDH* Isocitrate dehydrogenase, *LGG* Low-grade glioma, *LI* Labeling index, *MGMT* O-6-Methylguanine-DNA methyltransferase, *M* MGMT promoter methylation, *U* MGMT promoter unmethylation, *TERT* Telomerase reverse transcriptase

### Predictive efficacy of ITSS grade in relation to histological grade and different molecular markers

**Fig. 4:**
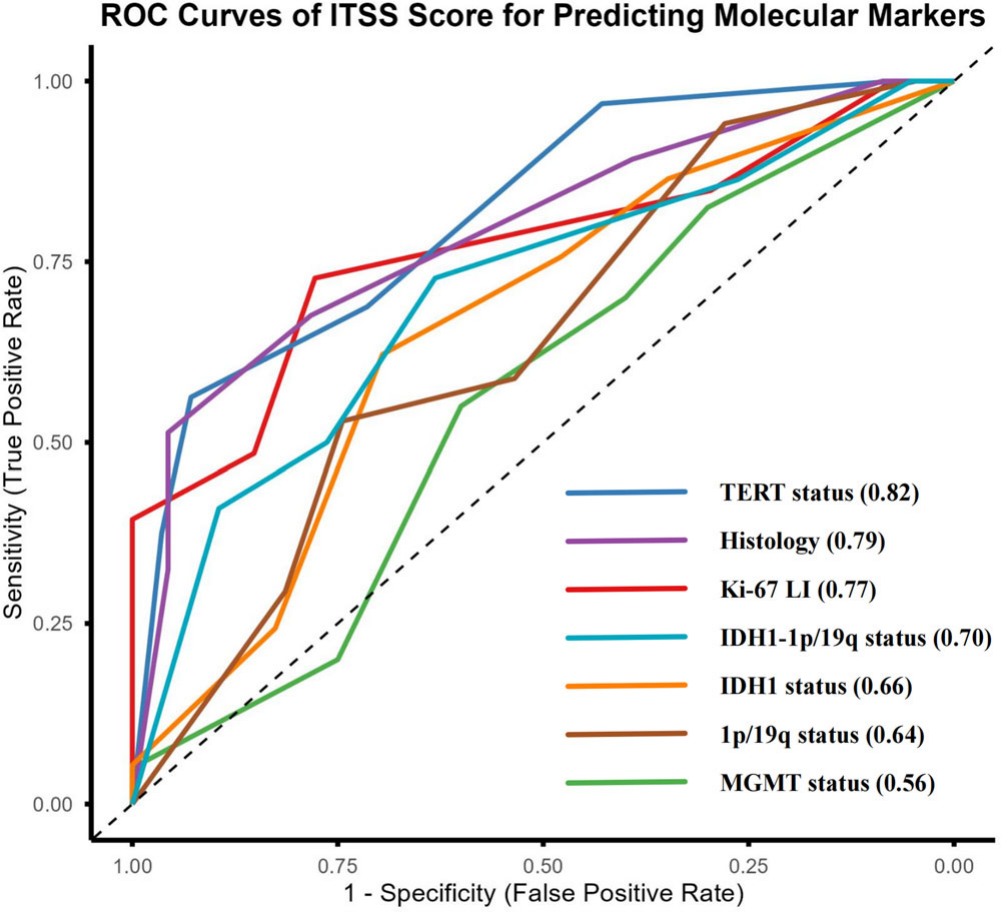
ROC curves were used to assess the role of ITSS grade in predicting histological grade and molecular biomarkers

ROC analysis demonstrated that ITSS grade discriminated histologic grade, Ki-67 expression, and TERT promoter mutation status with moderate to good performance (Fig. 4 and Table 3). For prediction of high *versus* low histologic grade, an ITSS cutoff of 2.5 yielded a sensitivity of 67.6%, a specificity of 78.3%, and an AUROC of 0.79. For prediction of high *versus* low Ki-67 expression, the same cutoff of 2.5 achieved a sensitivity of 72.7%, a specificity of 77.8%, and an AUROC of 0.77. For the prediction of TERT mutation status, an ITSS cutoff of 2.5 resulted in a sensitivity of 68.8%, a specificity of 71.4%, and the highest AUROC of 0.82. ITSS grade did not predict IDH1 mutation, MGMT promoter methylation, or 1p/19q co-deletion status with statistically significant accuracy.

**Table 3:** AUROC, optimal cutoff values, specificity (%), and sensitivity (%) for glioma histological grading, Ki-67 LI, TERT, MGMT, IDH1, and 1p/19q status

	AUROC	Cutoff	Spe (%)	Sen (%)
TERT status	0.82	2.5	71.4%	68.8%
Histology	0.79	2.5	78.3%	67.6%
Ki-67 LI	0.77	2.5	77.8%	72.7%
IDH1-1p/19q status	0.70	2.5	63.2%	72.7%
IDH1 status	0.66	2.5	69.6%	62.2%
1p/19q status	0.64	3.5	74.4%	52.9%
MGMT status	0.56	2.5	60.0%	55.0%

*AUROC* Area under the receiver operating characteristic curve, *IDH* Isocitrate dehydrogenase, *MGMT* O-6-Methylguanine-DNA methyltransferase, *Sen* Sensitivity, *Spe* Specificity, *TERT* Telomerase reverse transcriptase

Because IDH1 mutation and 1p/19q co-deletion define key molecular subtypes, we performed a *post hoc* analysis that combined IDH1 status and 1p/19q co-deletion into a single variable to identify IDH1–wild-type gliomas without 1p/19q co-deletion. For this unfavorable molecular subtype, ROC analysis again identified an ITSS cutoff of 2.5, which yielded a sensitivity of 72.7%, a specificity of 63.2%, and an AUROC of 0.70 (Fig. 4).

Multivariate logistic regression assessed the relationships between ITSS grade and molecular markers and quantified the independent contribution of each marker (Table 4). Ki-67 LI and TERT mutation status emerged as independent predictors of ITSS grade. High Ki-67 expression associated strongly with higher ITSS grade (OR = 11.69, 95% confidence interval 3.20–42.67), and TERT mutation also showed a strong positive association with ITSS grade (OR = 15.14, 95% confidence interval 4.49–51.02). Forest plots in Supplementary Fig. S2 visualize these associations and their confidence intervals.

**Table 4:** Analysis of molecular markers associated with glioma ITSS grading based on univariate and multivariate logistic regression

Variables	Single factor					Multifactor (all factors)				
	β	S.E	t	*p*-value	OR (95% CI)	β	S.E	t	*p*-value	OR (95% CI)
Sex										
Female					1.00 (Reference)					
Male	-0.00	0.46	-0.01	0.994	1.00 (0.41–2.45)					
Age	0.06	0.02	3.00	**0.003**	1.06 (1.02–1.11)	0.07	0.02	2.78	**0.006**	1.07 (1.02–1.12)
Histology										
LGG					1.00 (Reference)					1.00 (Reference)
HGG	2.06	0.54	3.84	**< 0.001**	7.82 (2.73–22.37)	1.17	0.67	1.74	0.081	3.22 (0.87–11.97)
Ki-67 LI										
Low					1.00 (Reference)					1.00 (Reference)
High	1.85	0.51	3.61	**< 0.001**	6.33 (2.33–17.25)	2.46	0.66	3.72	**< 0.001**	11.69 (3.20–42.67)
IDH1 status										
Wild-type					1.00 (Reference)					1.00 (Reference)
Mutant	-1.07	0.49	-2.18	**0.029**	0.34 (0.13–0.90)	0.89	0.64	1.40	0.160	2.45 (0.70–8.52)
TERT status										
Non-mutant					1.00 (Reference)					1.00 (Reference)
Mutant	2.28	0.54	4.24	**< 0.001**	9.79 (3.41–28.11)	2.72	0.62	4.38	**< 0.001**	15.14 (4.49–51.02)
1P/19q status										
Non-codeletion					1.00 (Reference)					
Codeletion	-0.88	0.51	-1.72	0.086	0.42 (0.15–1.13)					
MGMT status										
Non-methylation					1.00 (Reference)					
Methylation	-0.41	0.50	-0.82	0.411	0.66 (0.25–1.76)					

Bold values indicate statistical significance *p* < 0.05

IDH1 status “+”: IDH1 mutation; IDH1 status “-”: no IDH1 mutation

Low expression: Ki-67 LI < 10%; high expression: Ki-67 LI ≥ 10%

TERT status “+”: TERT promoter mutation; TERT status “-”: no TERT promoter mutation

*CI* Confidence intervals, *HGG* High-grade glioma, *ITSS* Intratumoral susceptibility signals, *IDH* Isocitrate dehydrogenase, *LGG* Low-grade glioma, *LI* Labeling index, *MGMT* O-6-Methylguanine-DNA methyltransferase, *M* MGMT promoter methylation, *U* MGMT promoter unmethylation, *OR* Odds ratios, *TERT* Telomerase reverse transcriptase

### Path analysis of ITSS grade and molecular markers

**Fig. 5:**
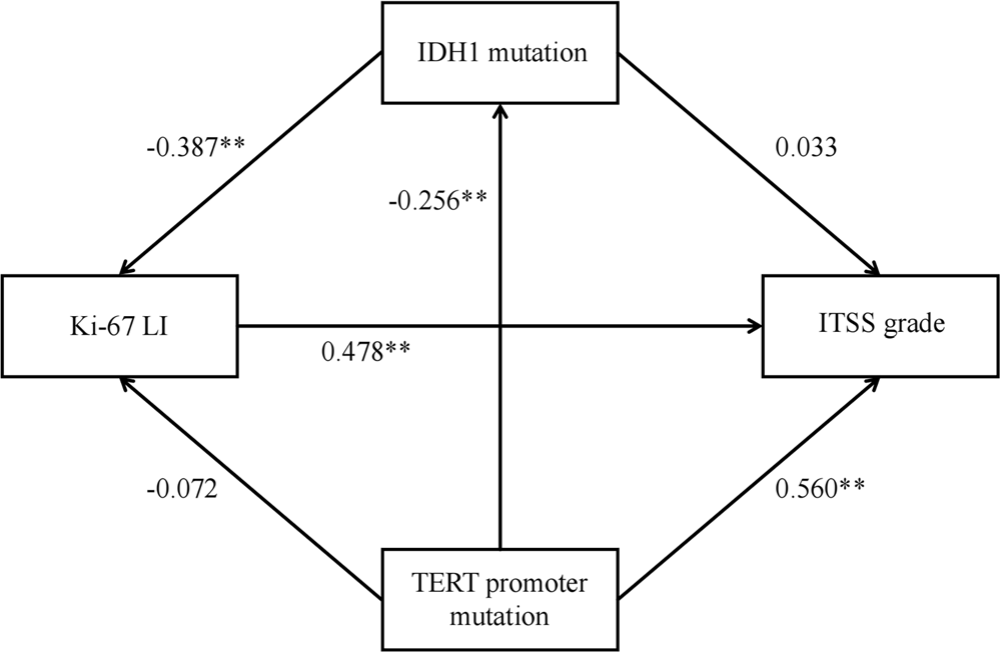
Path analysis diagram of the relationship between ITSS grade and molecular markers. The arrow (→) indicates the path influence relationship. The numbers in the figure represent standardized path coefficients, and the asterisks (*) indicate significance (* *p* < 0.05, ** *p* < 0.01). IDH, Isocitrate dehydrogenase; ITSS, Intratumoral susceptibility signals; LI, Labeling index; TERT, Telomerase reverse transcriptase

To further examine the interrelationship among ITSS grade and molecular markers, we conducted a structural model and performed path analysis, using recommended fit indices for evaluation [21]. The model fit the data well, the detailed path coefficients are presented in Table 5, the corresponding path diagram is shown in Fig. 5, and the model fit parameters are shown in Supplementary Table S4.

**Table 5:** Regression coefficients in the path analysis model of the relationship between ITSS grade and molecular markers

X	→	Y	SP	SE	z	*p*-value
TERT mutation	→	IDH1 mutation	-0.256	0.122	-2.053	**0.040**
TERT mutation	→	Ki-67 LI	-0.072	0.123	-0.585	0.559
IDH1 mutation	→	Ki-67 LI	-0.387	0.127	-3.124	**0.002**
TERT mutation	→	ITSS grade	0.560	0.275	6.141	**< 0.010**
IDH1 mutation	→	ITSS grade	0.033	0.303	0.335	0.737
Ki-67 LI	→	ITSS grade	0.478	0.287	5.047	**< 0.010**

Bold values indicate statistical significance *p* < 0.05

The arrow (→) indicates the path influence relationship

*ITSS* Intratumoral susceptibility signals, *IDH* Isocitrate dehydrogenase, *LI* Labeling index, *SP* Standardized path coefficients, *TERT* Telomerase reverse transcriptase

Standardized path coefficients showed significant positive effects of TERT mutation on ITSS grade (standardized path coefficient = 0.560, *p* < 0.010) and Ki-67 LI on ITSS grade (standardized path coefficient = 0.478, *p* < 0.010). IDH1 mutation correlated negatively with Ki-67 LI (standardized path coefficient = -0.387, *p* = 0.002). Although paths between IDH1 mutation and ITSS grade, and between TERT mutation and Ki-67 LI appeared in the model, these paths did not reach statistical significance. Taken together, these results indicate that Ki-67 LI and TERT mutation influence ITSS grade largely through independent direct effects, without substantial confounding from the other molecular markers. The negative association between IDH1 mutation and Ki-67 LI, combined with the direct effect of Ki-67 LI on ITSS grade, suggests that Ki-67 LI mediates the impact of IDH1 mutation on ITSS grade.

## Discussion

The glioma microenvironment provides a phenotypic readout of underlying tumor biology and holds significant clinical value when linked to molecular pathology through imaging [22]. In this study, we combined ultra-high field 7-T SWI, with an in-plane resolution of 100 µm, and molecular profiling to probe this relationship. We observed that gliomas with higher ITSS grades more often exhibited high histologic grade, elevated Ki-67 LI (≥ 10%), and TERT promoter mutation, and typically showed an IDH1–wild-type profile. ITSS grade predicted histologic grade, Ki-67 LI, and TERT status with moderate to good accuracy. Path analysis further indicated that Ki-67 LI and TERT mutation exerted direct effects on ITSS grade, whereas IDH1 mutation influenced ITSS grade indirectly through Ki-67 LI. These findings position ITSS grade as a simple, visually derived biomarker that links microvascular susceptibility signals with key molecular drivers of glioma aggressiveness.

SWI-derived ITSS already contributes to clinical glioma management by informing preoperative grading, differentiating calcification from microbleeds, characterizing abnormal vasculature, aiding differential diagnosis, and monitoring treatment response [23–25]. Numerous studies have confirmed that ITSS distribution corresponds to regions of high vascular density and perfusion on dynamic contrast-enhanced MRI and to histologic hotspots of neovascularization [17, 26]. Building on the 3-T ITSS grading standard [17], we optimized the 7-T ITSS grade criteria in combination with the distribution characteristics of the data and the imaging manifestations. The new grading scheme not only expanded the number of grading levels but also, in combination with the advantage of 7-T SWI in displaying richer ITSS signals, increased the grading thresholds at different levels to more accurately reflect the detection capability of ITSS by 7-T SWI. For example, small hemorrhages or vascular abnormalities that may be overlooked in low-field SWI can be clearly displayed in 7-T SWI. The assessment of ITSS grade by the two neuroradiologists was highly consistent, with grading discrepancies observed in only 5% of cases.

In terms of preoperative grading, HGG in our cohort consistently showed a higher ITSS grade than LGG. This pattern likely reflects the accumulation of microbleeds and abnormal neovascularization that accompanies more aggressive tumor behavior [27]. Chuang et al found that patients with high-grade astrocytoma contained more intratumoral ITSS signals than low-grade astrocytoma counterparts [28]. Bhattacharjee et al demonstrated significant differences in ITSS-related vascular volume across glioma grades using R2*-based quantitative methods [25]. Together with our findings, these studies support ITSS as a robust imaging biomarker for glioma grade. Clinically, ITSS grading may provide an objective preoperative indicator that helps refine surgical planning, guide the extent of resection, and inform decisions about adjuvant radiotherapy and chemotherapy.

The malignant evolution of gliomas depends strongly on specific molecular alterations [29]. In our study, glioma patients with high Ki-67 expression had higher ITSS grade. Ki-67 LI serves as a key marker for glioma growth kinetics and prognosis [30]. A recent study showed that gliomas with high Ki-67 expression showed more necrosis, edema, ill-defined margins, and vascular enhancement, and also higher ITSS grades than tumors with low Ki-67 expression [9]. This also corroborates our results and suggests that high Ki-67 LI reflects active tumor cell proliferation, invasive growth, and increased angiogenesis, which may increase the risk of tumor bleeding.

IDH1 mutation is a common molecular event in gliomas. In our cohort, IDH1 mutant gliomas showed lower ITSS grades than IDH1 wild-type gliomas, consistent with previous studies [14, 31]. Mechanically, IDH1 mutation can activate hypoxia-inducible factor‒HIF-1α signaling, alter energy metabolism, and upregulate the expression of vascular endothelial growth factor‒VEGF [32–34]. However, Grewal et al found a negative association between IDH mutation and vascular endothelial growth factor expression [35]. Our data, together with these prior findings, support the notion that IDH1-mutant tumors form fewer or less abnormal vessels than IDH1–wild-type tumors, despite the complex upstream signaling. Epigenetic modifications, including histone and DNA hypermethylation, as well as microenvironmental interactions, may contribute to this paradox by blunting angiogenic output. Path analysis also suggests that IDH1 mutations reduce Ki-67 LI, which in turn lowers on ITSS grade. Previous studies have also reported lower Ki-67 expression in IDH1-mutant gliomas, a phenomenon possibly linked to a more “quiescent” immune and stromal milieu [36]. Further in-depth analysis from the perspective of pathophysiological mechanisms is still needed in the future.

TERT promoter mutations represent another key determinant of glioma biology. Tumors harboring this mutation often show elevated TERT mRNA and telomerase activity, which sustain telomere length and promote unchecked proliferation [37]. Studies have linked TERT promoter mutations to WHO grade and adverse prognosis, underscoring their role in glioma progression [38]. In our study, TERT-mutant gliomas displayed higher ITSS grades than TERT-wild-type tumors, suggesting more microbleeds and denser or more abnormal vasculature. This pattern may reflect structurally atypical vessels with endothelial cell proliferation and incomplete basement membranes, which destabilize the vascular wall and favor leakage and hemorrhage [39]. Although prior studies have not systematically examined the relationship between TERT status and ITSS, advanced MRI techniques have already shown promise in TERT-related imaging. Zhang et al found that histogram analysis of dynamic contrast-enhanced MRI parameters predicted TERT mutation status and prognosis [40], and Park et al used diffusion and perfusion MRI to predict TERT mutation status of IDH wild-type LGGs [41]. Both studies collectively demonstrated the validity and potential of MRI in glioma research.

Our *post hoc* analysis further showed that ITSS grade could predict molecular subtype characterized by IDH wild-type and absence of 1p19q co-deletion, a profile characteristic of primary glioblastoma (GBM), the most common and highly aggressive malignant brain tumor in adults [42]. Higher ITSS grade in this subtype aligns with high tumor heterogeneity, abnormal neovascularization, and frequent microhemorrhages that typify GBM [43, 44]. It is worth noting that there may be a more complex relationship between 1p/19q status and ITSS, which needs to be further investigated in larger-scale studies with comprehensive molecular subtype stratification of patients.

Growing evidence supports multiparametric MRI, including structural, perfusion, diffusion, and metabolic sequences, as a powerful tool for noninvasive grading and molecular profiling of gliomas [9, 31, 45]. This study extended the literature by demonstrating that a single 7-T SWI sequence, interpreted through a refined ITSS grading system, can predict histologic grade and selected molecular markers. Beyond preoperative assessment, the link between ITSS grade and postoperative molecular markers also carries value. ITSS may forecast invasive phenotypes and vascular hyperactivity at recurrence, influence sensitivity to therapies that target angiogenesis or hypoxia [46], and provide biologically informed endpoints for optimizing SWI acquisition and postprocessing strategies, including texture and habitat analysis.

Our study also has some limitations. First, the retrospective nature of our study meant that preoperative CT scans were not routinely available for all patients, so we could not definitively exclude calcifications using the standard imaging modality. Second, SWI imaging features are related to the amount of intratumoral bleeding, and small amounts of bleeding may interfere with low signal areas, thereby masking other structures within the lesion. Although the high resolution of 7 T can reduce this kind of impact to some extent. Third, the sample size remained modest because 7-T MRI still sees limited clinical use, and the ITSS cutoff values that we derived from this cohort therefore require validation in larger, independent datasets. Lastly, ITSS grading relies on semiquantitative visual assessment rather than fully quantitative SWI metrics. To reduce subjectivity, we used blinded independent readings and expert arbitration, but subtle reader bias may persist.

Despite these limitations, our study still holds important clinical implications for the future. Noninvasive prediction models that integrate radiomics, deep learning, and multimodal MRI continue to gain traction for decoding glioma heterogeneity and enabling more precise preoperative subtyping [47]. Within this framework, SWI and ITSS can function as a bridge between *in vivo* imaging and molecular pathology, linking microvascular architecture and microhemorrhages to genetic and epigenetic alterations. Automated extraction and analysis of ITSS features using artificial intelligence may further improve diagnostic efficiency and accuracy. Quantitative susceptibility mapping (QSM) could add an objective estimate of tissue magnetic susceptibility and help validate or refine the qualitative ITSS-based assessments performed in this study. In addition, combining 7-T SWI with other advanced modalities, such as MR spectroscopy, perfusion-weighted imaging, and diffusion-weighted imaging, may ultimately yield integrated prediction models that more comprehensively capture glioma molecular features and improve noninvasive classification.

In conclusion, 7-T SWI-derived ITSS grade has the potential to predict the histological grade, Ki-67 LI, and TERT mutation status of gliomas, and shows promise as a noninvasive imaging biomarker that complements conventional diagnosis and prognostic tools. Moreover, Ki-67 LI and TERT mutation exert relatively independent effects on ITSS grade, while IDH1 mutation affects ITSS grade indirectly through its impact on Ki-67 LI. This may reveal the differential mechanisms of various molecular pathological factors in tumor microvascular architecture, providing a new perspective for understanding the biological basis behind the imaging phenotype.

## Supplementary information

**Additional file 1**: **Supplementary Table S1**. ITSS four-grade classification scheme. **Supplementary Table S2**. Distribution of ITSS four-grade classification scheme. **Supplementary Table S3**. Clinical and SWI characteristics of glioma patients with different molecular status. **Supplementary Table S4**. Parameter table for evaluation of fit of path analysis structural model. **Supplementary Fig. S1**. Appearance of ITSS in gliomas at different field strengths. 3-T SWI (left) and 7-T SWI (right). By comparing the two images, it can be seen that subtle lesions such as microbleeds or vascular abnormalities may be overlooked in 3T SWI, but can be clearly visualized on 7-T SWI. **Supplementary Fig. S2**. Frequency distribution of intratumoral susceptible signals (ITSS) counts. This histogram shows the distribution of ITSS counts in 60 glioma patients in our cohort. The data distribution shows four natural peaks, and the thresholds selected by the six-level ITSS grade system (10, 35, 50, and 85 ITSS, respectively) are set at the peaks. These thresholds are set at the natural inflection points of the data distribution to divide the population into groups with different ITSS signals. **Supplementary Fig. S3**. Use of phase images for differentiating calcifications. These are the SWI images and phase images of a patient with a glioma classified as ITSS grade 3. (**a**) The SWI image shows a hypointense lesion resembling microbleeds within the tumor (arrow) and hypointense bands representing neovascularization (triangle). (**b**) The corresponding SWI phase image shows the same lesion with low signal (arrow), which is opposite to the venous signal and is consistent with the characteristics of calcification. This lesion was therefore excluded from the ITSS count. The neovascularization appears as a moderately hyperintense band (triangle). This example demonstrates how concurrent phase image analysis was utilized to distinguish calcifications from paramagnetic blood products during ITSS grade. **Supplementary Fig. S4**. Forest plot of logistic regression for independent predictors of ITSS grade in gliomas. CI, confidence intervals; HGG, high-grade glioma; ITSS, intratumoral susceptibility signals; IDH, Isocitrate dehydrogenase; +, IDH1 mutation; -, no IDH1 mutation; LGG, low-grade glioma; low expression, Ki-67 LI < 10%; high expression, Ki-67 LI ≥ 10%; LI, labeling index; MGMT, O-6-methylguanine-DNA methyltransferase; M, MGMT promoter methylation; U, MGMT promoter unmethylation; OR, odds ratios; TERT, Telomerase Reverse Transcriptase; +, TERT promoter mutation; -, no TERT promoter mutation.

## Data Availability

The datasets used and analysed during the current study are available from the corresponding author on reasonable request.

## Abbreviations

HGG: High-grade glioma
IDH1: Isocitrate dehydrogenase 1
ITSS: Intratumoral susceptibility signals
LGG: Low-grade glioma
LI: Labeling index
MGMT: O-6-methylguanine-DNA-methyltransferase
MRI: Magnetic resonance imaging
OR: Odds ratio
ROC: Receiver operating characteristic
SWI: Susceptibility-weighted imaging
TERT: Telomerase reverse transcriptase
WHO: World Health Organization
