# Supplementary material for: 7-T MRI intratumoral susceptibility signals reflect biomarker status in gliomas
Source: Eur Radiol Exp. 2026 Apr 2;10:40. doi: 10.1186/s41747-026-00696-0 (PMC13047024; doi:10.1186/s41747-026-00696-0)
Supplement: Supplementary file 1 — Additional file 1: Supplementary Table S1. ITSS four-grade classification scheme. Supplementary Table S2. Distribution of ITSS four-grade classification scheme. Supplementary Table S3. Clinical and SWI characteristics of glioma patients with different molecular status. Supplementary Table S4. Parameter table for evaluation of fit of path analysis structural model. Supplementary Fig. S1. Appearance of ITSS in gliomas at different field strengths. 3-T SWI (left) and 7-T SWI (right). By comparing the two images, it can be seen that subtle lesions such as microbleeds or vascular abnormalities may be overlooked in 3T SWI, but can be clearly visualized on 7-T SWI. Supplementary Fig. S2. Frequency distribution of intratumoral susceptible signals (ITSS) counts. This histogram shows the distribution of ITSS counts in 60 glioma patients in our cohort. The data distribution shows four natural peaks, and the thresholds selected by the six-level ITSS grade system (10, 35, 50, and 85 ITSS, respectively) are set at the peaks. These thresholds are set at the natural inflection points of the data distribution to divide the population into groups with different ITSS signals. Supplementary Fig. S3. Use of phase images for differentiating calcifications. These are the SWI images and phase images of a patient with a glioma classified as ITSS grade 3. (a) The SWI image shows a hypointense lesion resembling microbleeds within the tumor (arrow) and hypointense bands representing neovascularization (triangle). (b) The corresponding SWI phase image shows the same lesion with low signal (arrow), which is opposite to the venous signal and is consistent with the characteristics of calcification. This lesion was therefore excluded from the ITSS count. The neovascularization appears as a moderately hyperintense band (triangle). This example demonstrates how concurrent phase image analysis was utilized to distinguish calcifications from paramagnetic blood products during ITSS grade. Supplementary [file 41747_2026_696_MOESM1_ESM.pdf]

7-T MRI intratumoral susceptibility signals reflect biomarker status in gliomas

ELECTRONIC SUPPLEMENTARY MATERIAL

MRI protocol

The MRI protocol included T1-seighted Magnetization prepared 2 rapid acquisition gradientecho–MP2RAGE (repetition time/echo time = 5,000 ms / 2 ms, with a voxel size of 0.65 × 0.65 × 0.65 mm^3, bandwidth = 240Hz/Px, flip angle = 4°/4°, inversion time = 1,000 ms / 3,200 ms); T2-weighted turbo spin-echo (repetition time/echo time = 10,000/43 ms, with a voxel size of 0.3 × 0.3 × 3.0 mm^3, bandwidth = 240Hz/Px, flip angle = 135°); and SWI using a three-dimensional fully flow-compensated gradient echo sequence and reconstructed SWI by combining magnitude and phase images. SWI parameters included repetition time/echo time = 21/14 ms, voxel size of 0.1 × 0.1 × 1.5 mm^3, bandwidth = 210Hz/Px, flip angle = 10°.

Instructions for the formulation of grading criteria

Previous studies have proved that Local magnetic field perturbations caused by susceptibility differences increase linearly with static magnetic field strength (B0), while the signal-to-noise ratio (SNR) is proportional to the square of B0 ($SNR \propto B0^2$) [1]. These field-strength effects can increase conspicuity of susceptibility-related structures at 7 T, but they can also amplify B0 inhomogeneity and susceptibility-induced signal attenuation. To derive a clinically practical and reliable 7-T ITSS grade standard, we first anchored our approach to established 3-T four-level grading scheme and increased the thresholds to account for the higher ITSS burden typically visible at 7 T. Fig. S1 provides an illustrative 3-T *versus* 7-T comparison supporting this adjustment.

During development, we found that a four-level scheme compressed the observed ITSS counts at 7 T and failed to represent the distribution granularity within our cohort (Tables S1 and S2). We therefore expanded the number of grade levels and set the numerical thresholds using natural inflection points in the ITSS count distribution across the 60 included patients. Fig. S2 illustrates this distribution and the locations of the inflection points that guided threshold selection.

Detailed information on the experimental methods of molecular pathology detection

Detection of Ki-67 labeling index. Resected tumor tissue was fixed in 10% neutral formalin for 24 hours and processed routinely for paraffin embedding. We performed Ki-67 immunohistochemical using the Envision two-step method and MIB-1 monoclonal antibody(Dako, diluted 1:100), followed by DAB chromogenic visualization. We selected five random high-power fields at 400x magnification and counted at least1000 tumor cells. We reported Ki-67 labeling index (LI) as the percentage of tumor cell nuclei showing positive staining among the total counted nuclei. We dichotomized Ki-67 expression using a 10% cutoff, defining LI ≥ 10% as high expression and LI < 10% as low expression [2].

Detection of IDH1 mutation status. We screened for the IDH1 R132H mutation using immunohistochemistry with the mutation-specific monoclonal antibody H09 (Dianova; diluted 1:50). We defined positive status by brown cytoplasmic and/or nucleus staining. For cases negative on immunohistochemistry, we sequenced by exon 4 of the IDH1 gene using Sanger sequencing. We used the following primers: forward 5'-CGGTCTTCAGAGAAGCCATT-3' and reverse 5'-GCAAAATCACATTATTGCCAAC-3'.

Detection of 1p/19q co-deletion. We assessed 1p/19q status using dual-color fluorescence in situ hybridization (FISH) with probes for 1p36/1q25 and 19q13/19p13 (Vysis), following the manufacturer's protocol. We counted 100 tumor cell nuclei per case. We defined deletion when the target-to-control signal ratio was ≤0.8. We defined co-deletion when both 1p and 19q met deletion criteria.

Detection of TERT promoter mutation. We extracted genomic DNA from formalin-fixed paraffin-embedded (FFPE) tissue and amplified the TERT promoter region covering C228T and C250T hotspots by PCR. We performed Sanger sequencing on PCR products to identify C228T (c.-124C > T) and C250T (c.-146C > T) mutations. We used the following primers: forward 5'-CAGCGCTGCCTGAAACTC-3' and reverse 5'-GTCCTGCCCCTTCACCTT-3'.

Detection of MGMT promoter methylation. We evaluated MGMT promoter methylation using Methylation-specific PCR (MSP). We bisulfite-treated extracted DNA and amplified the converted DNA using methylation- and unmethylation-specific primer sets. We used the following methylated-specific primers: forward 5'-TTTCGACGTTCGTAGGTTTTCGC-3' and reverse 5'-GCACTCTTCCGAAAACGAAACG-3'. We included fully methylated CpG DNA as a positive control and normal brain tissue DNA as a negative control. We defined MGMT promoter methylation as positive when electrophoresis demonstrated a methylation-specific band after PCR.

References

1. Enjilela R, MacMillan B, McAloon MJ, Petrov OV, Vashaee S, Balcom BJ (2019) Controlling susceptibility mismatch effects, signal lifetimes, and SNR through variation of B(0) in MRI of rock core plugs. J Magn Reson 307 :106575 https://doi.org/10.1016/j.jmr.2019.106575
2. Yang X, Hu C, Xing Z et al (2023) Prediction of Ki-67 labeling index, ATRX mutation, and MGMT promoter methylation status in IDH-mutant astrocytoma by morphological MRI, SWI, DWI, and DSC-PWI. Eur Radiol 33(10):7003-7014 https://doi.org/10.1007/s00330-023-09695-w

Supplementary Table S1. ITSS four-grade classification scheme

Grades	Number
Grade 0	No ITSS
Grade I	1-10
Grade II	10-35
Grade III	>35

ITSS, intratumoral susceptibility signals

Supplementary Table S2. Distribution of ITSS four-grade classification scheme

Grades	Number of patients
Grade 0	2
Grade I	11
Grade II	17
Grade III	30

As shown in Supplementary Table 2, according to our original ITSS four-grade classification scheme, the population is largely concentrated in Grade III, which fails to reveal the detailed distribution of ITSS numbers obtained at 7 T.

Supplementary Table S3. Clinical and SWI characteristics of glioma patients with different molecular status

Characteristics	Histology			IDH1status			Ki-67 LI		
	**LGG**	**HGG**	***p-value***	+	−	***p-value***	**Low**	**High**	***p-value***
**Age**	46.41±10.49	49.97±12.12	0.235	47.14±10.10	51.00±13.47	0.134	48.89±11.05	48.44±12.18	0.727
**Sex(males/females)**	13/10	14/23	0.157	18/19	9/14	0.471	14/13	13/20	0.335
**ITSS grade**			**<0.001**			**0.04**			**<0.001**
**ITSS 0**	2	0		2	0		2	0	
**ITSS 1**	7	4		7	4		6	5	
**ITSS 2**	9	8		14	3		13	4	
**ITSS 3**	5	25		14	16		6	24	
**Characteristics**	**MGMT status**			**1p/19q status**			**TERT status**		
	**M**	**U**	***p-value***	**Codeletion**	**Non-codeletion**	***p-value***	+	−	***p-value***
**Age**	49.49±10.95	47.00±12.83	0.599	50.35±8.97	47.95±12.51	0.445	51.00±11.11	45.85±11.69	0.072
**Sex(males/females)**	19/21	8/12	0.582	8/9	19/24	0.840	16/16	11/17	0.405
**ITSS grade**			0.547			0.173			**0.001**
**ITSS 0**	2	0		0	2		0	2	
**ITSS 1**	5	5		1	10		1	10	
**ITSS 2**	14	3		6	11		9	8	
**ITSS 3**	18	12		10	20		22	8	

Binary data are shown as counts; age is expressed as mean ± standard deviation; HGG, high-grade glioma; ITSS, intratumoral susceptibility signals; IDH, Isocitrate dehydrogenase; +, IDH1 mutation; -, no IDH1 mutation; LGG, low-grade glioma; low expression, Ki-67 LI < 10%; high expression, Ki-67 LI ≥ 10%; LI, labeling index; MGMT, O-6-methylguanine-DNA methyltransferase; M, MGMT promoter methylation; U, MGMT promoter unmethylation; TERT, Telomerase Reverse Transcriptase; +, TERT promoter mutation; -, no TERT promoter mutation;.

Supplementary Table S4. Parameter table for evaluation of fit of path analysis structural model

Test parameters	Parameter values
GFI	1.0
NFI	1.0
CFI	1.0
IFI	1.0
RMSEA	0

CFI, Comparative Fit Index; GFI, Goodness of Fit Index; IFI, Incremental Fit Index; NFI, Normed Fit Index; RMSEA, Root Mean Square Error of Approximation

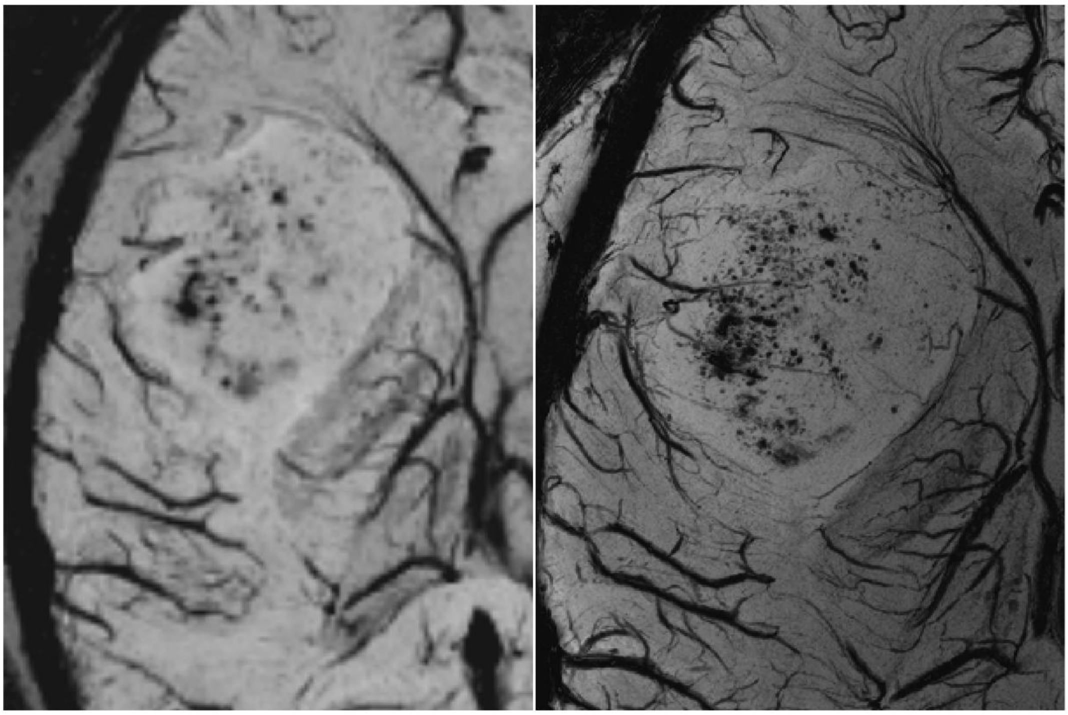

Supplementary Fig. S1. Appearance of ITSS in gliomas at different field strengths. 3-T SWI (left) and 7-T SWI (right). By comparing the two images, it can be seen that subtle lesions such as microbleeds or vascular abnormalities may be overlooked in 3T SWI, but can be clearly visualized on 7-T SWI.

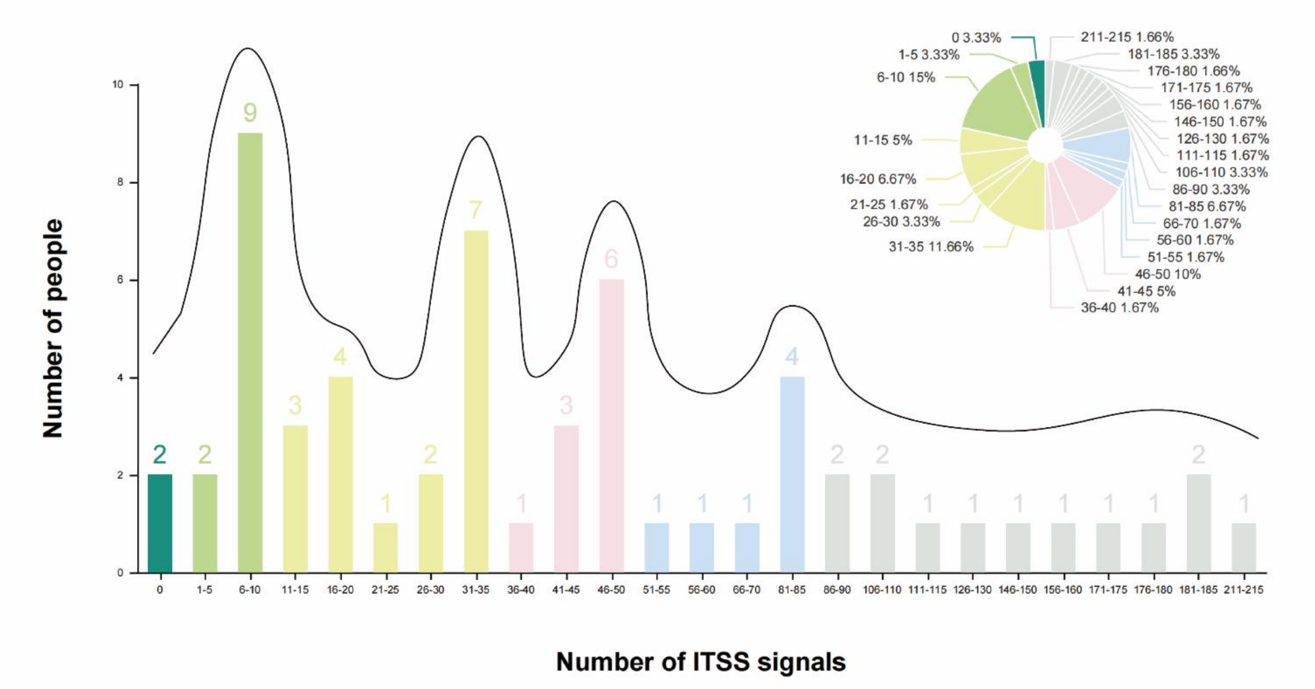

Supplementary Fig. S2. Frequency distribution of intratumoral susceptible signals (ITSS) counts. This histogram shows the distribution of ITSS counts in 60 glioma patients in our cohort. The data distribution shows four natural peaks, and the thresholds selected by the six-level ITSS grade system (10, 35, 50, and 85 ITSS, respectively) are set at the peaks. These thresholds are set at the natural inflection points of the data distribution to divide the population into groups with different ITSS signals.

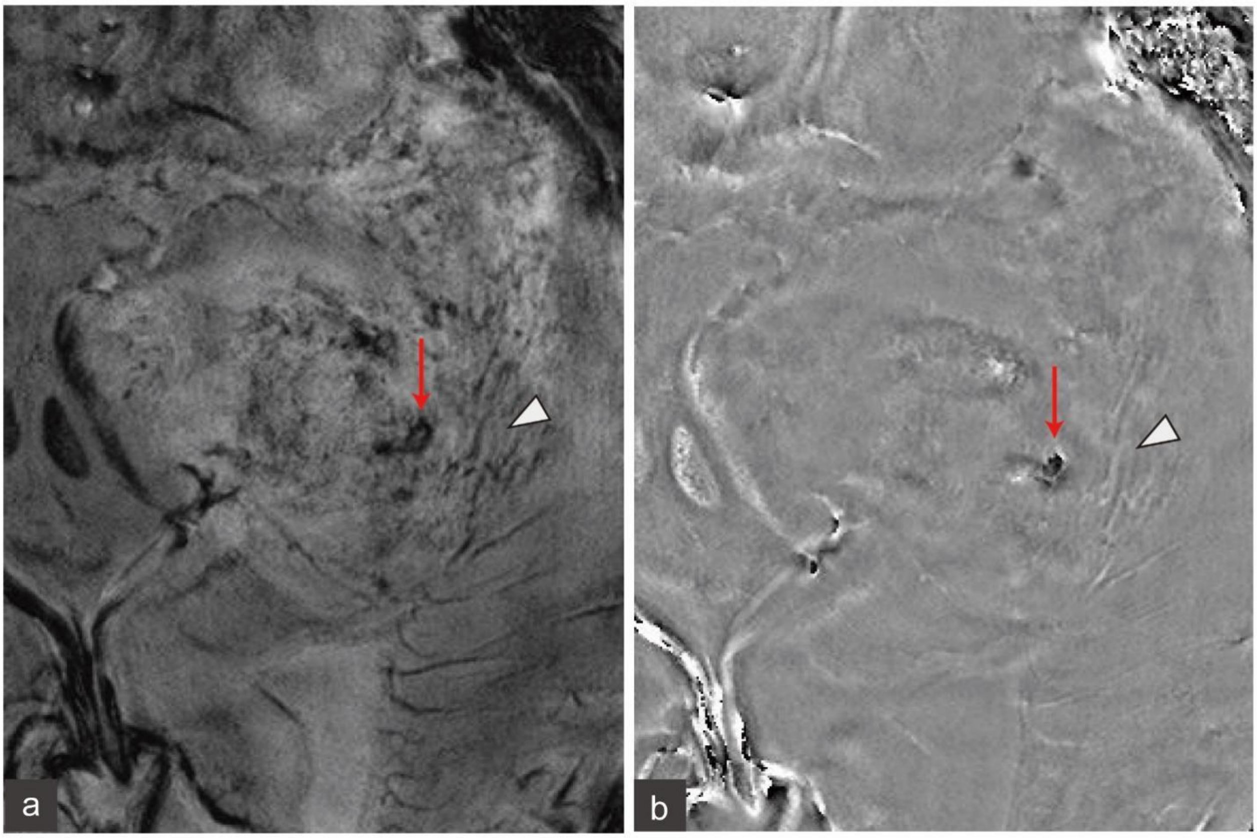

Supplementary Fig. S3. Use of phase images for differentiating calcifications. These are the SWI images and phase images of a patient with a glioma classified as ITSS grade 3. (**a**) The SWI image shows a hypointense lesion resembling microbleeds within the tumor (arrow) and hypointense bands representing neovascularization (triangle). (**b**) The corresponding SWI phase image shows the same lesion with low signal (arrow), which is opposite to the venous signal and is consistent with the characteristics of calcification. This lesion was therefore excluded from the ITSS count. The neovascularization appears as a moderately hyperintense band (triangle). This example demonstrates how concurrent phase image analysis was utilized to distinguish calcifications from paramagnetic blood products during ITSS grade.

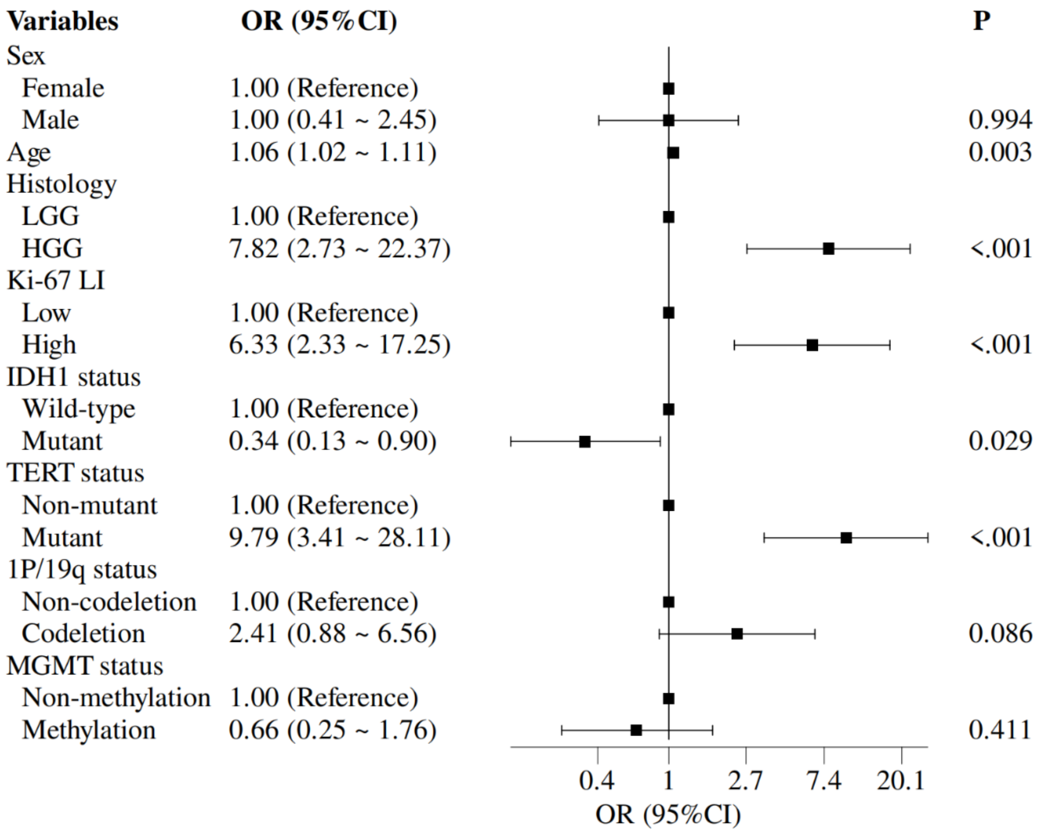

Supplementary Fig. S4. Forest plot of logistic regression for independent predictors of ITSS grade in gliomas.

CI, confidence intervals; HGG, high-grade glioma; ITSS, intratumoral susceptibility signals; IDH, Isocitrate dehydrogenase; +, IDH1 mutation; -, no IDH1 mutation; LGG, low-grade glioma; low expression, Ki-67 LI<10%; high expression, Ki-67 LI ≥ 10%; LI, labeling index; MGMT, O-6-methylguanine-DNA methyltransferase; M, MGMT promoter methylation; U, MGMT promoter unmethylation; OR, odds ratios; TERT, Telomerase Reverse Transcriptase; +, TERT promoter mutation; -, no TERT promoter mutation.
